# Factors Influencing Astigmatic Correction Using Small-Incision Lenticule Extraction: A Systematic Review and Meta-Analysis

**DOI:** 10.1155/joph/5518587

**Published:** 2025-11-30

**Authors:** Shuangcheng Li, Xuan Chen, Jiaxin Song, Yimei Han, Yan Wang

**Affiliations:** ^1^School of Medicine, Nankai University, Tianjin, China; ^2^Clinical College of Ophthalmology, Tianjin Medical University, Tianjin, China; ^3^Tianjin Key Lab of Ophthalmology and Visual Science, Tianjin Eye Institute, Tianjin Eye Hospital, Nankai University Affiliated Eye Hospital, Tianjin, China; ^4^Nankai University Eye Institute, Nankai University, Tianjin, China

## Abstract

**Purpose:**

To systematically review SMILE-based astigmatism correction and influencing factors.

**Methods:**

Literature was screened across eight databases. Pre- and post-SMILE cylinder, difference vector (DV), correction index (CI), magnitude of error (ME), angle of error (AE), and index of success (IOS) were compared. Bias was assessed using Cochrane's Risk of Bias, Quality Assessment of Diagnostic Accuracy Studies, and the Newcastle–Ottawa Scale.

**Results:**

Elevated ocular residual astigmatism (ORA) resulted in greater postoperative residual astigmatism, accompanied by increased DV and IOS (*p* < 0.05), whereas ME, AE, and CI remained unaffected by ORA levels. Postoperative cylinder, DV, ME, AE, CI, and IOS were comparable between eyes (*p* > 0.05). Correction outcomes were impacted by ocular rotation, astigmatism characteristics, spherical degree, corneal curvature, and patient age.

**Conclusions:**

SMILE effectively corrects low, moderate, and high astigmatism, but high ORA patients tend to experience undercorrection. But accuracy requires vector planning.

## 1. Introduction

Small-incision lenticule extraction (SMILE) was introduced by Shah et al. in 2008 [1, 2]. It has been increasingly performed recently. SMILE has similar or better safety, efficacy, and predictability than laser-assisted in situ keratomileusis [3–9]. It is a flapless surgery with incisions of approximately 2–4 mm, resulting in fewer postoperative dry eyes [10–12], less induction of high-order aberrations [13], and reduced corneal inflammation and damage [14, 15]. SMILE maintains the integrity of the anterior 1/3 of the corneal stroma, improving biomechanical strength postoperatively [16–21].

However, the Food and Drug Administration-approved laser platform for SMILE (VisuMax, Carl Zeiss Meditec, Germany) lacks an eye-tracking system. Owing to the ability of the eye to rotate from a sitting to a supine posture, the doctor's experience, and the patient's cooperation [22–24], the precision of SMILE for correcting myopic astigmatism has caught extensive attention from clinical practitioners and scholars. Astigmatism is a vector requiring simultaneous consideration of its size and axis during refractive surgery. Compared to simple myopia correction, astigmatism is more prone to undercorrection or overcorrection and involves various complex factors [25]. Surgical outcomes may be influenced by high ocular residual astigmatism (ORA), which comprises posterior corneal, lenticular, and retinal astigmatism. These elements can disrupt the compensatory relationship between corneal and ORA, potentially reducing the accuracy of astigmatic correction. In addition to ORA, several other factors have been identified as potential contributors to surgical variability, including eye laterality [26–30], corneal curvature [31–33], corneal cap thickness [34–37], degree of myopia [38–40], laser energy [41], balanced salt solution irrigation [42], degree of ocular rotation [43, 44], incision location [45, 46], incision number [47, 48], incision size [49], optical zone diameter [50], age [51], ablation ratio [52], surgical plan [53], astigmatism correction plan [29], decentration [54], astigmatism type [55, 56], and learning curve [57]. Furthermore, correcting high astigmatism exhibits low predictability and stability [58–60], increasing the difficulty in precise astigmatism correction using SMILE.

No meta-analysis or systematic review has specifically targeted factors influencing astigmatism correction using SMILE. Therefore, this study aimed to review relevant research on the factors affecting astigmatism correction using SMILE and perform a meta-analysis and systematic review of these factors. The study could provide insight into developing new strategies for guiding clinical practitioners in comprehensively considering the factors influencing astigmatism correction, enhancing surgical precision, and achieving optimal patient satisfaction.

## 2. Methods

The meta-analysis was completed in concordance with the PRISMA statement (PROSPERO registration number: CRD42023466690; available from https://www.crd.york.ac.uk/prospero/display_record.php?ID=CRD42023466690).

### 2.1. Search Strategy

We searched eight databases, including Embase, PubMed, Cochrane Library, Web of Science, CNKI, VIP, CBM, and Wang Fang, for relevant literature published up to January 1, 2024. Free-text search terms included “SMILE,” “Small-incision lenticule extraction,” “Femtosecond laser small-incision lenticule extraction,” “Small-incision lenticule extraction,” “Refractive lenticule extraction,” “Femtosecond lenticule extraction,” “Small-incision corneal lens extraction,” “Lenticule extraction,” “corneal stromal lenticule,” “Astigmatism,” “Cylind,” “Vector analysis,” and “Astigmia.” The Medical Subject Headings terms included “Astigmatism.” Following the search, we reviewed the reference lists of the articles and added more pertinent research. Two researchers used Endnote 20.2.1 to select all included articles through the following steps: (1) title and abstract and (2) full text. Supporting Information (available here) provides the search strategy for PubMed.

### 2.2. Inclusion and Exclusion Criteria

The inclusion criteria included (1) study design: randomized cohort and case series studies, (2) patients with astigmatism without eye disease, and (3) SMILE.

The exclusion criteria included (1) research conducted in vitro; (2) meta-analyses and reviews; (3) case reports; (4) mixed astigmatism, hyperopia, and presbyopia; (5) current clinical trials and conference abstracts; and (6) absence of control or comparator interventions.

### 2.3. Outcome Parameter

Target-induced astigmatism (TIA): Changes in astigmatism were intended to be induced by surgery. Surgically induced astigmatism (SIA): Changes in astigmatism were actually induced by surgery. Difference vector (DV): The vectorial difference between TIA and SIA, representing the residual postoperative astigmatism (DV = TIA − SIA). Magnitude of error (ME): The arithmetic difference between the magnitudes of SIA and TIA. Angle of error (AE): The angle between the axes of TIA and SIA. Correction index (CI): The ratio of SIA to TIA (CI = |SIA|/|TIA|). CI > 1 indicates overcorrection, CI < 1 indicates undercorrection. Index of success (IOS): The ratio of the DV to TIA (IOS = |DV|/|TIA|), reflecting the proportion of residual astigmatism. Flattening index (FI): The effective component of the astigmatic correction along the intended meridian, calculated as FI = |SIA| × cos(AE)/|TIA| [61].

### 2.4. Data Extraction and Risk Assessment

Every study was screened and evaluated independently by two researchers based on the inclusion and exclusion criteria. In case of any doubts, a consensus was reached by reading the full text, followed by a discussion or decision of a third researcher. Two researchers independently extracted the data from the included studies, including the first author, publication date, country/region, study design type, baseline characteristics of the study participants, intervention measures, and relevant outcome indicators. Cochrane's Risk of Bias (ROB) [62], Quality Assessment of Diagnostic Accuracy Studies [63], and Newcastle–Ottawa Scale [64] were used to evaluate the methodological quality of the corresponding types of original studies. Two researchers conducted quality assessments separately, and any disagreements were settled by talking to or consulting a third researcher.

### 2.5. Statistical Analysis

Data analysis was performed using Cochrane Review Manager (RevMan version 5.4). The weighted mean differences and 95% confidence intervals were calculated for continuous outcomes. Heterogeneity was assessed using the Q-test and *I*^2^-test. An *I*^2^ value > 50% was considered statistically significant. A random-effects model was used because it is less influenced by the weight of each study [65]. A fixed-effects model was used when the heterogeneity level was < 50%. A two-tailed test with a *p*-value < 0.05 was considered statistically significant.

## 3. Results

**Figure 1:**
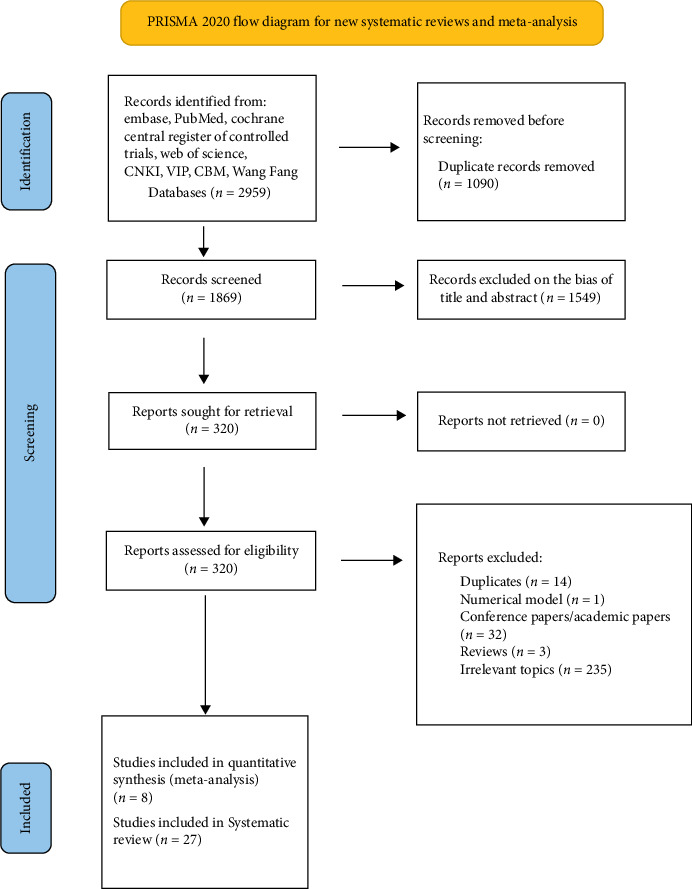
Flow diagram of the identification and inclusion of eligible studies.

We found 2959 possibly related articles from the eight databases; 1869 articles were available after the removal of duplicates using EndNote. Thirty-six research articles were eventually included after titles, abstracts, and full texts were screened; eight were used in the meta-analysis. The meta-analysis included one randomized, one cohort, and six case–control studies. The detailed selection process is illustrated in Figure 1.

**Figure 2:**
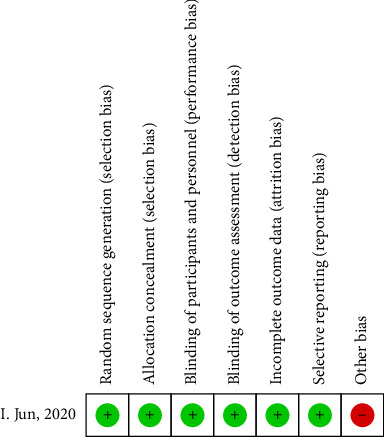
Risk of bias assessment of randomized controlled trials included.

Seven hundred and twenty-two eyes were included in the quality assessments and study characteristics for this meta-analysis.  Table 1 summarizes these characteristics. The ROB for the randomized controlled trial is shown in Figure 2. The risk of displayed bias was unknown owing to incomplete knowledge regarding sequence generation, allocation concealment, performance bias, and detection bias. The ROB for the case series studies is presented in Table 2, with all three high-quality articles. The ROB for cohort studies is shown in Table 3, with the total scores of the four high-quality articles being ≥ 9 points.

**Figure 3:**
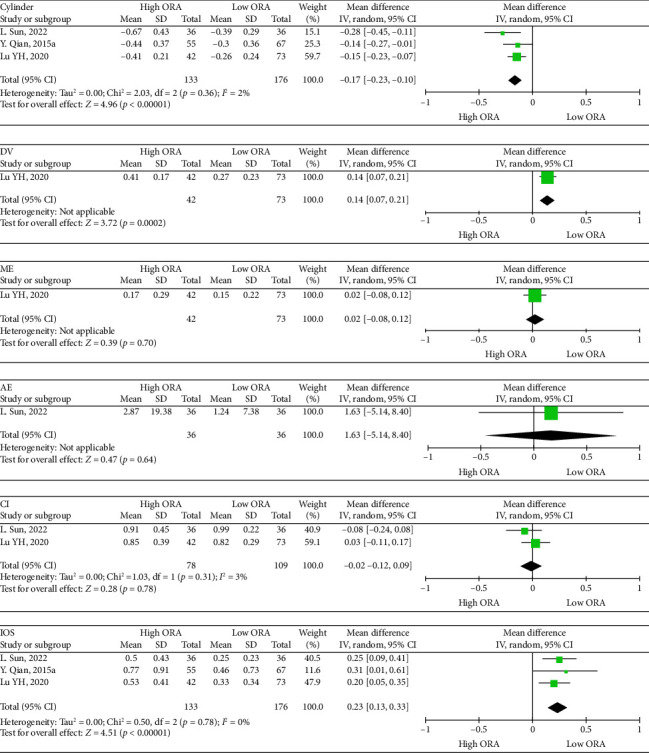
Forest plots showing the comparison of cylinder and efficacy index between the high ORA group and the low ORA group. ORA, ocular residual astigmatism; C: cylinder; DV: difference vector; ME: magnitude of error; AE: angle of error; CI: correction index; IOS: index of success; SD, standard deviation; CI, confidence interval; df, degrees of freedom; *I*^2^, extent of inconsistency; Z, overall effect.

**Figure 4:**
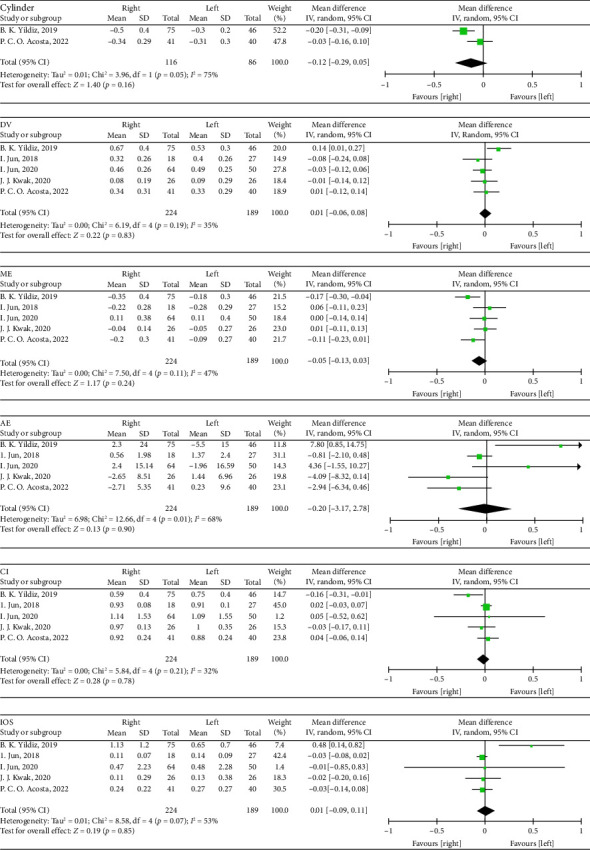
Forest plots showing the comparison of cylinder and efficacy index between right and left eyes. C: cylinder; DV: difference vector; ME: magnitude of error; AE: angle of error; CI: correction index; IOS: index of success; SD, standard deviation; CI, confidence interval; df, degrees of freedom; *I*^2^, extent of inconsistency; Z, overall effect.

1. The impact of ORA on the precision of astigmatism correction after SMILE [66–68]. Three studies were included for a meta-analysis comparing the effect of high (> 1.00 D) and low ORAs (≤ 1.00 D) on post-SMILE astigmatism correction (Figure 3).
    Cylinder: High ORA was −0.14 higher than low ORA (95% CI: −0.23–-0.10, *p*=<0.00001, Chi^2^ = 2.03, *I*^2^=2%).
    DV: High ORA was 0.14 higher than low ORA (95% CI: 0.07–0.21, *p*=0.0002).
    ME: The difference between high ORA and low ORA was not statistically significant (95% CI: −0.08–0.12, *p*=0.7).
    AE: The difference between high ORA and low ORA was not statistically significant (95% CI: −5.14–8.4, *p*=0.64).
    CI: The difference between high ORA and low ORA was not statistically significant (95% CI: −0.12–0.09, *p*=0.78, Chi^2^ = 1.03, *I*^2^=3%).
    IOS: High ORA was 0.23 higher than low ORA (95% CI: 0.13–0.33, *p* < 0.00001, Chi^2^ = 0.50, *I*^2^=0%).
2. Effect of eye laterality on the precision of astigmatism correction after SMILE [26–30]: Five studies were included in a meta-analysis comparing astigmatism correction outcomes between the eyes following SMILE (Figure 4).
    Cylinder: The difference between the right and left eyes was not statistically significant (95% CI: −0.29–0.05, *p*=0.16, Chi^2^ = 3.96, *I*^2^=75%).
    DV: The difference between the right and left eyes was not statistically significant (95% CI: −0.06–0.08, *p*=0.83, Chi^2^ = 6.19, *I*^2^=35%).
    ME: The difference between the right and left eyes was not statistically significant (95% CI: −0.13–0.03, *p*=0.24, Chi^2^ = 7.50, *I*^2^=47%).
    AE: The difference between the right and left eyes was not statistically significant (95% CI: −3.17–2.78, *p*=0.9, Chi^2^ = 12.66, *I*^2^=68%).
    CI: The difference between the right and left eyes was not statistically significant (95% CI: −0.07–0.06, *p*=0.78, Chi^2^ = 5.84, *I*^2^=32%).
    IOS: The difference between the right and left eyes was not statistically significant (95% CI: −0.09–0.11, *p*=0.85, Chi^2^ = 8.58, *I*^2^=53%).

We conducted a systematic review (Table 4) on the impact of additional factors influencing SMILE outcomes. First, ocular parameters—including corneal curvature [31–33], degree of myopia [38–40], type of astigmatism [55, 56], age [51], and degree of ocular rotation [43, 44]—can all affect surgical results. For instance, low corneal curvature may lead to undercorrection, and SMILE tends to yield better outcomes in correcting regular rather than irregular astigmatism [31–33]. Age-related increases in corneal stiffness may also reduce the effectiveness of astigmatic correction [51].

Second, surgical design and procedural parameters—such as corneal cap thickness [34–37], incision location and size [45, 46, 49], optical zone diameter [50], and laser energy settings [41]—play important roles. Thicker caps and smaller incisions, as in microincision lenticule extraction (MILE), have been associated with smoother healing and fewer postoperative aberrations [49]. Finally, other influencing factors such as surgical planning [53], balanced salt solution irrigation [42], and the surgeon's learning curve [57] also impact the stability and predictability of SMILE outcomes.

## 4. Discussion

This study conducted a meta-analysis and systematic review of the factors influencing SMILE astigmatism correction. Meta-analyses were performed on ORA [66–68] and eye laterality [26–30]. Patients with a high ORA (> 1.00 D) showed higher cylinder, DV, and IOS values post-SMILE correction than those with a low ORA (≤ 1.00 D), suggesting the need for preoperative surgical design adjustments according to vector planning to improve correction outcomes in patients with high ORA. Regarding eye laterality, the meta-analysis results showed no significant differences in astigmatism correction between the eyes.

Recent studies [4–6, 9, 69] have reported the long-term effects of SMILE on astigmatism correction. SMILE has been shown to be effective in correcting mild to high astigmatism [27, 70–73]. However, multiple factors may influence its effectiveness. Qian et al. [68] found a correlation between a high ORA and increased postoperative residual astigmatism. Moreover, the high ORA group exhibited a higher IOS than the low ORA group within 1–6 months postoperatively, implying reduced precision in correcting high ORA myopic astigmatism using SMILE. Lu et al. [66, 67] achieved similar results. Addressing and correcting ORAs on the anterior corneal surface may render the corneal surface overly irregular, resulting in higher-order aberrations and impacting postoperative vision. Compared to manifest refraction planning, vector planning for patients with a high ORA improved the precision of astigmatic correction [29]. Kwak et al. [27–30] found no significant differences because of eye laterality, which is consistent with our meta-analysis results. In contrast, Yildiz et al. [26] observed better correction in the left eye than in the right eye, perhaps due to better astigmatism correction from the superior incision as opposed to the temporal incision in the right eye.

Factors related to the patient's condition, including corneal curvature [31–33], degrees of myopia [38–40], degrees of eye rotation [43, 44], astigmatism type [55, 56], and age [51], were also discussed. The SMILE procedure followed the curvature principle rather than the flattening principle during the docking process. An ideal contact surface conforms perfectly to the corneal surface; however, for patients with astigmatism, the varying steepness and flatness along the meridian can affect the efficacy of SMILE astigmatism correction, potentially resulting in undercorrection for patients with low curvature (≤ 41 D) [31–33]. Compared to that of correcting low-to-moderate myopia, the predictability of visual acuity for correcting high myopia using SMILE is lower [38–40]. Astigmatism correction involves a vector, and precise correction along the expected correcting axis is crucial, particularly in highly astigmatic eyes. Axial errors are often caused by eye rotation and are divided into static and dynamic rotations [74, 75]. Eye rotation occurs while standing or sitting in the supine position (static rotation), whereas involuntary eye movement during surgery represents dynamic rotation. The SMILE negative-pressure suction system can correct dynamic eye rotation relatively well; however, in the studies included, eye rotation did not affect the efficacy of SMILE astigmatism correction, possibly because the studies had slight rotation angles [43, 44]. Swami et al. [76] proposed that a 4° rotation may result in 14% undercorrection, a 6° rotation in 20% undercorrection, and a 16° rotation in > 50% undercorrection. Concerning different astigmatism correction types, studies [55, 56] found that SMILE has better correction effects on regular astigmatism. Age is also crucial to astigmatism correction efficacy because corneal stromal stiffness increases with age. Therefore, SMILE may have poorer outcomes in older patients [51].

Factors related to surgical design include corneal cap thickness [34–37], incision location [45, 46], incision number [47, 48], incision size [49], laser energy [41], balanced salt solution irrigation [42], optical zone diameter [50], ablation ratio [52], decentration [54], and astigmatism correction plan [29]. In surgical parameters, surgeons can alter the corneal cap thickness to within 100–160 μm. Studies have compared the impact of thin and thick corneal caps on postoperative outcomes. Thicker corneal caps of 120 μm resulted in reduced corneal healing responses, smoother lenticules, and better recovery outcomes [37]. However, some studies [34–37] presented conflicting results. Concerning incision positions and numbers, although no standard incision size for SMILE surgery exists, the commonly recommended angles are 120° and 90°, and other angles are used to avoid conjunctival vessels with one to two incisions [47, 48]. SMILE typically involves 2–4 mm incisions; however, ongoing research has reduced the incision size to 2 mm, termed MILE surgery [49]. In studies by Tian et al. [77] and Wang et al. [49], smaller incisions induced fewer aberrations, leading to minimal postoperative astigmatism fluctuations, suggesting that MILE offers good predictability and stability.

Other factors potentially affecting astigmatism correction were studied, such as the use of balanced salt solution irrigation postsurgery, where significant intergroup differences were detected in equivalent spherical and cylindrical values at 1 day and 1 month compared with using plain water. However, long-term follow-up showed no appreciable variations between the two groups [42]. Another study focused on laser energy and spacing. When the spot distance was too large for the selected energy setting, the residual corneal stroma required mechanical separation, leading to increased irregularity. However, if the spot spacing is too small for the selected energy level, the combining cavitation bubbles could affect the next few laser beams and result in more imperfections on the surface [41]. Using two different laser settings to create lenticules (standard mode: 140 nJ, spot distance 3.0 mm; fast mode: 170 nJ, spot distance 4.5 mm) is currently recommended. Different decentration and ablation ratios do not affect SMILE astigmatism correction; however, larger amounts may increase postoperative higher-order aberrations [52, 54].

In addition, other factors, such as surgical planning [53] and learning curve [57], have been studied. Different surgical plans (same day/elective) showed no significant differences in astigmatism correction using SMILE [53]. The varying skill levels of surgeons may affect the accuracy of astigmatism correction in both eyes. Although studies have demonstrated the high effectiveness, safety, and predictability of SMILE in correcting myopic and astigmatism, inexperienced ophthalmologists undergo an early learning phase, following which the effectiveness of surgery gradually improves [57].

In conclusion, SMILE is widely used for astigmatism correction, but residual astigmatism is more common in patients with high ORA. The degree of eye rotation, astigmatism magnitude and type, spherical degree, corneal curvature, and other factors influenced astigmatism correction. Due to potential biases in the included studies, several limitations should be noted. First, the number of randomized controlled trials (RCTs) was limited, particularly those evaluating post-SMILE astigmatism correction using vector analysis. Second, most included studies were published in English or Chinese, which may have introduced publication bias. Third, variations in follow-up durations among studies may have influenced the outcomes. Lastly, although standardized mean difference (SMD) was used to reduce measurement bias across different scales, it may have affected the precision of the pooled estimates. Although SMILE is now one of the mainstream surgical options for corneal refractive correction, further studies are required to assess the clinical effectiveness of SMILE. The findings of the study could help improve astigmatism correction and patients' quality of life.

## Figures and Tables

**Table 1:** Main characteristics of the included studies.

Study (first author, year)	Lu et al. [66]		Sun et al. [67]		Qian et al. [68]		Jun et al. [29]		Kwak et al. [27]		Acosta et al. [30]		Yildiz et al. [26]		Jun et al. [28]	
Study design	Cohort study		Cohort study		Case series		RCT		Case series		Cohort study		Case series		Case series	
N (eyes)	42	73	36	36	55	67	64	50	26	26	41	40	46	75	18	27
Age (mean ± SD)	/	/	27.75 ± 7.57	27.17 ± 7.08	26.04 ± 6.79	27.4 ± 6.68	/	/	/	/	31.59 ± 0.92	30.5 ± 0.96	/	/	/	/
SE (mean ± SD)	−6.05 ± 2.14	−5.95 ± 2.06	/	/	/	/	/	/	/	/	−5.48 ± 1.56	−5.76 ± 1.68	/	/	/	/
Group	High ORA (≥ 1.00 D)	Low ORA (< 1.00 D)	High ORA (> 1.0 D)	Low ORA (≤ 1.0 D)	High ORA (> 1.0 D)	Low ORA (≤ 1.0 D)	Right	Left	Right	Left	Right	Left	Left	Right	Right	Left
Preoperatively C (mean)	−1.09	−0.98	−1.64	−1.87	−0.7	−0.76	/	/	/	/	−1.43	−1.4	−1.4	−1.39	/	/
Preoperatively C (SD)	0.58	0.49	0.8	0.92	0.53	0.72	/	/	/	/	0.75	0.66	0.6	0.65	/	/
Preoperatively C (min)	0	0	−0.25	−0.75	/	/	/	/	/	/	−0.75	−0.75	−1	−1	/	/
Preoperatively C (max)	−3	−3	−3.5	−4	/	/	/	/	/	/	−3.25	−3	/	/	/	/
Follow-up	3 months	3 months	12 months	12 months	6 months	6 months	6 months	6 months	6 months	6 months	3 months	3 months	3 months	3 months	6 months	6 months
Postoperatively C (mean)	−0.41	−0.26	−0.67	−0.39	−0.44	−0.3	/	/	/	/	−0.34	−0.31	−0.3	−0.5	/	/
Postoperatively C (SD)	0.21	0.24	0.43	0.29	0.37	0.36	/	/	/	/	0.29	0.3	0.2	0.4	/	/
TIA (mean)	1.11	1	/	/	/	/	0.96	1.01	1.27	1.23	1.44	1.41	1.3	1.3	2.96	2.86
TIA (SD)	0.6	0.51	/	/	/	/	0.54	0.51	0.82	0.75	0.77	0.68	0.5	0.5	0.44	0.41
SIA (mean)	0.94	0.85	/	/	/	/	1.07	1.11	1.23	1.18	1.24	1.33	1.17	0.9	2.73	2.58
SIA (SD)	0.61	0.54	/	/	/	/	0.57	0.59	0.82	0.74	0.68	0.76	0.5	0.5	0.43	0.41
DV (mean)	0.41	0.27	/	/	/	/	0.46	0.49	0.08	0.09	0.34	0.33	0.53	0.67	0.32	0.4
DV (SD)	0.17	0.23	/	/	/	/	0.26	0.25	0.19	0.29	0.31	0.29	0.3	0.4	0.26	0.26
ME (mean)	0.17	0.15	/	/	/	/	0.11	0.11	−0.04	−0.05	−0.2	−0.09	−0.18	−0.35	−0.22	−0.28
ME (SD)	0.29	0.22	/	/	/	/	0.38	0.4	0.14	0.27	0.3	0.27	0.3	0.4	0.28	0.29
|ME| (mean)	/	/	/	/	/	/	0.3	0.33	/	/	/	/	/	/	/	/
|ME| (SD)	/	/	/	/	/	/	0.25	0.25	/	/	/	/	/	/	/	/
AE (mean)	/	/	2.87	1.24	/	/	2.4	−1.96	−2.65	1.44	−2.71	0.23	−5.5	2.3	0.56	1.37
AE (SD)	/	/	19.38	7.38	/	/	15.14	16.59	8.51	6.96	5.35	9.6	15	24	1.98	2.4
|AE| (mean)	11.7	7.5	10.9	4.8	/	/	10.99	11.14	2.65	1.67	/	/	/	/	1.44	1.96
|AE| (SD)	15.4	12.4	16.2	5.7	/	/	10.6	12.36	8.36	6.9	/	/	/	/	1.42	1.93
CI (mean)	0.85	0.82	0.91	0.99	/	/	1.14	1.09	0.97	1	0.92	0.88	0.75	0.59	0.93	0.91
CI (SD)	0.39	0.29	0.45	0.22	/	/	1.53	1.55	0.13	0.35	0.24	0.24	0.4	0.4	0.08	0.1
IOS (mean)	0.53	0.33	0.5	0.25	0.77	0.46	0.47	0.48	0.11	0.13	0.24	0.27	0.65	1.13	0.11	0.14
IOS (SD)	0.41	0.34	0.43	0.23	0.91	0.73	2.23	2.28	0.29	0.38	0.22	0.27	0.7	1.2	0.07	0.09
FI (mean)	/	/	/	/	/	/	/	/	/	/	0.86	0.88	0.87	0.72	/	/
FI (SD)	/	/	/	/	/	/	/	/	/	/	/	/	/	/	/	/

*Note:* C: cylinder.

Abbreviations: AE, angle of error; CI: correction index; DV: difference vector; FI: flattening index; IOS: index of success; ME: magnitude of error; SIA: surgically induced astigmatism; TIA: target-induced astigmatism.

**Table 2:** Risk of bias assessment based on the Newcastle–Ottawa scale for case–control studies.

Study aspects	Criteria	Lu et al. [66]	Sun et al. [67]	Acosta et al. [30]
		Stars (points)		
Selection	Representativeness of the exposed cohort	1	1	1
	Selection of the nonexposed cohort	1	1	1
	Ascertainment of exposure	1	1	1
	Demonstration that outcome of interest was not present at the start of the study	1	1	1

Comparability	Comparability of cohorts on the basis of design or analysis	2	2	2

Outcome	Assessment of outcome	1	1	1
	Adequacy of follow-up duration	1	1	1
	Was follow-up long enough for outcome to occur	1	1	1

Total score		9	9	9

*Notes:* The total maximum achievable score was nine stars (or points), which reflects the highest methodological quality; higher scores (more stars) on the Newcastle–Ottawa scale suggest better methodological quality and a lower risk of bias in the study.

**Table 3:** Risk of bias assessment based on the quality assessment of diagnostic accuracy studies for cohort studies.

QUADAS criteria	Description	Y. Qian, 2015	B. K. Yildiz, 2019	J. J. Kwak, 2020	I. Jun, 2018
Patient selection	Representative selection of patients for the diagnostic test	Yes (1)	Yes (1)	Yes (1)	Yes (1)
	Justification of the study sample size	Yes (1)	Yes (1)	Yes (1)	Yes (1)

Index test	Appropriateness and reproducibility of the diagnostic test evaluation	Yes (1)	Yes (1)	Yes (1)	Yes (1)
	Time interval between index test and reference standard	Yes (1)	Yes (1)	Yes (1)	Yes (1)

Reference standard	Appropriateness and accuracy of the reference standard	Yes (1)	Yes (1)	Yes (1)	Yes (1)
	Application of the reference standard regardless of index test results	Yes (1)	No (0)	Yes (1)	No (0)

Flow and timing	Appropriate interval between index test and reference standard	Yes (1)	No (0)	Yes (1)	Yes (1)
	Completion of index test and reference standard for all patients	Yes (1)	Yes (1)	Yes (1)	Yes (1)

Interpretation of results	Interpretation of index test result without knowledge of reference standard results	Yes (1)	Yes (1)	Yes (1)	Yes (1)
	Prespecification of thresholds used in the study	Yes (1)	Yes (1)	Yes (1)	Yes (1)

Total		High (10)	High (8)	High (10)	High (9)

*Note:* The QUADAS consists of 10 items covering key domains such as patient selection, index test, reference standard, flow and timing, and interpretation of results. Each item was scored as 1 (yes) or 0 (no or unclear), with a maximum total score of 10. Based on the total scores, studies were categorized as high quality and low risk of bias (8–10 points), moderate quality (5–7 points), and low quality and high risk of bias (1–4 points).

**Table 4:** Factors influencing astigmatic correction using SMILE.

Study	Country	Research type	Factors	Study groups	Sample size	Astigmatism correction effect	Conclusion
Zhao [31]	China	Cohort study	Corneal curvature	Low corneal curvature (< 41 D)	42	Cylinder: −0.04 ± 0.02	SMILE has shown good improvements in vision for patients with different corneal curvature, and SMILE is safe and effective
				Moderate corneal curvature (41–46 D)	52	Cylinder: −0.04 ± 0.01	
				High corneal curvature (> 46 D)	50	Cylinder: −0.05 ± 0.02	

Liu et al.[32]	China	Cohort study	Corneal curvature	Low corneal curvature (≤ 41 D)	36	Cylinder: 0	SMILE offers safe and effective correction for myopic eyes with different corneal curvatures, and patients with low curvature exhibit mild “under-correction” within 1–2 mm corneal diameter
				Regular corneal curvature (41–46 D)	36	Cylinder: 0	
				High corneal curvature (≥ 46 D)	36	Cylinder: 0	

Luft et al. [33]	Austria Germany	Cohort study	Corneal curvature	Low corneal curvature (≤ 41 D)	21	Cylinder: −0.39 ± 0.26	SMILE induces more spherical aberrations in eyes with flat corneal curvature
				Regular corneal curvature (42.0–46.9 D)	21	Cylinder: −0.49 ± 0.26	
				High corneal curvature (≥ 47.0 D)	21	Cylinder: −0.62 ± 0.32	

Güell et al. [34]	Spain	NRCT	Corneal curvature	130 µm	44	Cylinder: −0.17 ± 0.36	Four different corneal cap thicknesses (130/140/150/160 μm) showed no differences in postoperative vision, optical visual quality, or complications. Furthermore, this study can serve as evidence for secondary surgery to remove small incision lenses postoperatively
				140 µm	14	Cylinder: −0.06 ± 0.12	
				150 µm	12	Cylinder: −0.1 ± 0.23	
				160 µm	24	Cylinder: −0.11 ± 0.47	

Lee et al. [35]	South Korea	Case series	Corneal curvature	120 µm	554	Cylinder: −0.13 ± 0.14	To get the same refractive results for varying cap thicknesses, the attempted correction is modified based on cap thickness
				130 µm	377	Cylinder: −0.13 ± 0.14	
				140 µm	90	Cylinder: −0.12 ± 0.14	

Liu et al. [36]	China	RCT	Corneal curvature	120 µm	40	Cylinder: −0.01 ± 0.04	Three months postoperatively, the thickness of the corneal cap does not affect visual outcomes
				140 µm	40	Cylinder: 0	

Gülmez et al. [37]	Turkey	Case series	Corneal curvature	120 µm	47	Cylinder: 0 ± 0.37	Caps with thicknesses of 120 and 140 μm are safe and effective for correcting myopia or myopic astigmatism. Patients with 140 μm corneal cap thickness showed greater improvement in uncorrected distance visual acuity (UDVA) after a 12-month follow-up compared to those with 120 μm
				140 µm	47	Cylinder: 0 ± 0.19	

Han et al. [38]	China	Case series	Myopic diopter	High myopia (−6 to −8.75 D)	40	Cylinder: −0.26 ± 0.25	SMILE is effective in correcting high myopia and myopic astigmatism. In the SMILE group, 12 eyes with correction inadequacy > 1.0 D in extremely high myopia (equal to or greater than −9 D) were observed, indicating a potential need for further nomogram modifications for the treatment of extremely high myopia
				Highly severe myopia (−9 to −12 D)	35	Cylinder: −0.48 ± 0.34	

Taneri et al. [39]	Germany	Case series	Myopic diopter	High myopia (−8 to −11.38 D)	53	Cylinder: −0.38 ± 0.24	SMILE corrects high myopia (−8 to −10 D) and moderate myopia (−3 to −8 D) similarly, with a maximum correction of approximately −3 D of astigmatism, yielding good visual and refractive outcomes.
				Moderate myopia (−3 to −8.88 D)	361	Cylinder: −0.26 ± 0.26	SMILE demonstrates effectiveness, safety, predictability, and stability in low, moderate, and high myopia. A trend of under-correction in the high myopia group suggests the need for nomogram adjustments

Torky and Alzafiri [40]	Kuwait	Case series	Myopic diopter	Low myopia (−1.00 to −3.00 D)	94	Cylinder: −0.5 ± 0.25	SMILE demonstrates effectiveness, safety, predictability, and stability in low, moderate, and high myopia. The trend of undercorrection in the high myopia group suggests the need for nomogram adjustments
				Moderate myopia (−3.25 to −6.00 D)	95	Cylinder: −0.5 ± 0.56	
				High myopia (−6.25 to −10.00 D)	85	Cylinder: −0.5 ± 0.44	

Kamiya et al. [41]	Japan	Case series	Energy settings	140 nJ (spot size 3.0 mm)	22	Cylinder: 0	SMILE with energy settings of 140 (spot size 3.0 mm) and 170 nJ (spot size 4.5 mm) are effective for correcting myopia and myopic astigmatism
				170 nJ (spot size 4.5 mm)	22	Cylinder: 0	

Liu et al. [42]	China	RCT	Balanced salt solution	Balanced salt solution	52	Cylinder: −0.1 ± 0.43	Significant intergroup differences were observed at 1 day and 1 month postoperatively in equivalent spherical and cylindrical lenses, but not in myopia correction. Its early visual outcomes are superior to traditional SMILE surgery
				Sterile water	52	Cylinder: 0.06 ± 0.53	

Wang et al. [43]	China	Cohort study	Eye rotation	Mild eye rotation (≤ 3°)	72	DV: 0.25(0.0.5)	Despite lacking an eye static rotation compensation system, active pupil tracking, topography, or aberrometry-guided features, SMILE demonstrates good astigmatism correction. This is facilitated by the negative pressure suction system and can effectively correct dynamic rotation
						ME: 0(0.0.25)	
						CI: 1(0.76.1)	
						IOS: 0.22(0.0.59)	
				Moderate to high eye rotation (> 3°)	56	DV: 0.25(0.0.5)	
						ME: 0.16(0.0.37)	
						CI: 0.88(0.68.1)	
						IOS: 0.28(0.0.53)	

Song et al. [44]	Korea	Cohort study	Eye rotation	Mild eye rotation (≤ 4°)	344	Cylinder: −0.09 ± 0.16	Eye rotation does not significantly affect the results of SMILE surgery, even in corrections > −1.50 D of astigmatism, where rotation does not influence residual astigmatism and uncorrected distance visual acuity (UCVA)
				High eye rotation (> 4°)	140	Cylinder: −0.11 ± 0.19	

Han et al. [46]	China	Cohort study	Incision positions	120∘	34	Cylinder: −0.24 ± 0.26	The incision location has an effect on the astigmatism effect; minimal astigmatism caused by surgical manipulation after 3 months was observed
						DV: 0.3 ± 0.25	
						ME: −0.1 ± 0.28	
						AE: 3.22 ± 21.62	
						CI: 0.97 ± 0.65	
						IOS: 0.47 ± 0.75	
				90∘	52	Cylinder: −0.16 ± 0.27	
						DV: 0.33 ± 0.28	
						ME: −0.21 ± 0.3	
						AE: 2.09 ± 15.77	
						CI: 0.9 ± 0.29	
						IOS: 0.35 ± 0.47	

Chan et al. [45]	China Hong Kong	Cohort study	Incision positions	Right temporal side	29	Cylinder: −0.28 ± 0.31	No significant differences in visual or refractive outcomes when the incision was made temporally or superiorly were observed
						DV: 0.27 ± 0.31	
						ME: 0.09 ± 0.17	
						AE: −6.9 ± 42.2	
						CI: 1.17 ± 0.43	
						IOS: 0.38 ± 0.95	
				Left superior side	29	Cylinder: −0.2 ± 0.24	
						DV: 0.2 ± 0.24	
						ME: 0.04 ± 0.21	
						AE: −0.39 ± 44.3	
						CI: 1.05 ± 0.52	
						IOS: 0.19 ± 0.83	

Moshirfar et al. [48]	USA	Cohort study	Number of incisions	Single incision	32	Cylinder: −0.38 ± 0.38	Single-incision SMILE is an effective and safe procedure that can improve vision in patients with myopia and astigmatism. With improved nomogram guidance and technological advancements, SMILE holds promise for enhancing clinical outcomes. In cases of regular astigmatism and higher preoperative cylinder power, undercorrection may occur, while in irregular astigmatism and lower preoperative cylinder power scenarios, overcorrection may occur
Abdelwahab et al. [47]	Egypt	Case series	Number of incisions	Bilateral incision	105	Cylinder: 0.2 ± 0.31	Bilateral-incision SMILE is an effective and safe approach that offers stable and predictable outcomes for correcting myopia and astigmatism. Additionally, the use of dual incisions allows better irrigation of the corneal stromal pockets
Wu et al. [50]	China	Cohort study	Optical zone	Small zone (6.1–6.4 mm)	21	Cylinder: −0.20 ± 0.3	SMILE improves the myopic patients' visual quality. The small aperture group showed significant postoperative astigmatism differences compared with preoperative levels, while the large aperture group exhibited no differences. This suggests that using a smaller optical zone during surgery only slightly affects nighttime vision
				Big zone (6.5–6.8 mm)	30	Cylinder: −0.15 ± 0.28	

Primavera et al. [51]	Spain	Case series	Age	≤ 35 years	51	Cylinder: −0.13 ± 0.26	Comparing patients aged ≤ 35 and ≥ 40, older patients had lower postoperative visual acuity compared with younger ones. The ≥ 40 age group showed lower uncorrected visual acuity (UCVA) and corrected distance visual acuity (CDVA) than the ≤ 35 age group. Although post-SMILE refractive results were acceptable, they were inferior to those of younger patients, showing lower efficacy and safety indices, poorer astigmatic outcomes, and a tendency for undercorrection, with a significantly lower proportion of postoperative astigmatism within ±0.5 D compared with the younger group. This may be attributed to increased corneal stromal hardness with age, leading to reduced corneal stromal remodeling capacity post-SMILE, thereby impacting the corrective effect
						DV: 0.13 ± 0.27	
						ME: 0.04 ± 0.23	
						AE: −0.67 ± 9.06	
						CI: 1.09 ± 0.42	
						IOS: 0.19 ± 0.44	
				≥ 40 years	51	Cylinder: −0.32 ± 0.41	
						DV: 0.38 ± 0.58	
						ME: 0.03 ± 0.43	
						AE: −4.05 ± 15.59	
						CI: 0.9 ± 0.58	
						IOS: 0.35 ± 0.68	

Wang et al. [49]	China	Case control	Incision size	MILE (2.0 mm)	41	Cylinder: −0.16 ± 0.21	No statistical significance between SMILE and MILE regarding equivalent sphere and cylinder degrees at 6 months postoperatively was observed. However, MILE showed smaller fluctuations in cylinder during recovery. Vector analysis results indicated smaller astigmatic vectors in the 45° and 135° directions in the MILE group. This may be owing to the approximately 30° incision angle in MILE compared with 50°–80° in SMILE, resulting in fewer changes in corneal anterior surface morphology during incision healing, thereby reducing astigmatism in oblique axes. MILE (1.5–2.0 mm or smaller) is safe and effective, exhibiting good predictability and stability, reducing astigmatism in oblique axes, preserving corneal tissue integrity and stability better, and resulting in fewer postoperative complications
				SMILE (3–5 mm)	41	Cylinder: −0.21 ± 0.28	

Kim et al. [53]	Korea	Cohort study	Surgical plan	Same-day surgery	83	Cylinder: −0.48 ± 0.25	Postoperative results of patients undergoing SMILE on the same day as planned SMILE treatment showed no significant differences. Patients on the day of surgery may undergo pupil dilation and other fundus examinations, which could potentially affect surgical outcomes. However, this did not affect the efficacy of postoperative astigmatism correction
				Planned surgery	80	Cylinder: −0.48 ± 0.3	

Jun et al. [29]	South Korea	RCT	Astigmatism correction plan	Manifest refraction plan	58	Cylinder: −0.22 ± 0.18	The effectiveness of preoperative apparent refraction and vector planning for correcting postoperative astigmatism in patients with ORA ≥ 0.75 D was assessed. Apparent refraction and vector planning in SMILE were effective for correcting myopic astigmatism and high ocular residual astigmatism. The visual outcomes were comparable. The vector plan showed significant reductions in postoperative refraction, corneal astigmatism, and internal astigmatism. Using the vector plan could enhance the postoperative refractive results of SMILE for high ocular residual astigmatism. Repeat refraction before surgery for high ORA could achieve better corrective outcomes
						DV: 0.43 ± 0.26	
						ME: 0.17 ± 0.35	
						AE: −2.78 ± 17.57	
						CI: 1.24 ± 1.54	
						IOS: 0.52 ± 2.45	
				Vector plan	56	Cylinder: −0.14 ± 0.16	
						DV: 0.52 ± 0.24	
						ME: 0.04 ± 0.41	
						AE: 3.84 ± 13.28	
						CI: 0.98 ± 1.57	
						IOS: 0.43 ± 2.03	

Ivarsen et al. [56]	Denmark	Case series	Astigmatism type	With-the-rule astigmatism	605	AE: −1.3 ± 12	Pre-correcting astigmatism by 1.00 D leads to an undercorrection error of approximately 0.15 D. The correction for against-the-rule astigmatism was approximately 0.35 D less effective than for with-the-rule astigmatism. Considering against-the-rule/with-the-rule astigmatism preoperatively may yield better results in correcting high cylinder degrees
				Against-the-rule astigmatism	131	AE: −0.8 ± 6	

Perez-Izquierdo et al. [55]	Spain	Cohort study	Astigmatism type	With-the-rule astigmatism	56	ME: −0.05 ± 0.23	After grouping patients based on astigmatism types (with-the-rule, against-the-rule, oblique astigmatism) and further classifying them by different magnitudes, for astigmatism < 1.50 D, correction using SMILE was feasible without adjustments. However, when astigmatism was ≥ 1.50 D, against-the-rule and oblique astigmatisms exhibited higher levels of under-correction. In the highly astigmatic group, against-the-rule astigmatism had an average undercorrection of (0.37 ± 0.35) D, oblique astigmatism showed an average under-correction of (0.13 ± 0.25) D, and with-the-rule astigmatism had an average under-correction of (0.11 ± 0.29) D
						AE: −1.19 ± 7.79	
				Oblique astigmatism	16	ME: 0.05 ± 0.18	
						AE: −0.6 ± 11.8	
				Against-the-rule astigmatism	33	ME: 0.03 ± 0.13	
						AE: 0.42 ± 2.446	

Chan et al. [57]	China Hong Kong	Cohort study	Learning curve	Initial surgery	100	Cylinder: −0.24 ± 0.33	One group consisted of the initial 100 patients that a surgeon performed surgery independently upon commencing, while the second group comprised the most recent 100 patients. The findings indicated that with an increase in surgical experience, faster visual recovery, improved safety, and more accurate astigmatism correction could be achieved
						DV: 0.26 ± 0.32	
						ME: −0.04 ± 0.3	
						AE: 10.1 ± 19.2	
				Recent surgery	100	Cylinder: −0.16 ± 0.23	
						DV: 0.16 ± 0.23	
						ME: −0.005 ± 0.19	
						AE: 5.4 ± 14.3	

Huang et al. [54]	China	Cohort study	Decentration	Decentration < 0.1 mm	23	Cylinder: −0.41 ± 0.3	No significant differences were observed among the postoperative predictability, corrective efficacy, safety, and visual outcomes among different amounts of decentration. However, for patients with high astigmatism, an increase in decentration resulted in increased postoperative spherical aberration and coma
				Decentration 0.1–0.2 mm	41	Cylinder: −0.26 ± 0.35	
				Decentration > 0.2 mm	50	Cylinder: −0.41 ± 0.36	

Li et al. [52]	China	Cohort study	Ablation depth ratio size	Ablation depth ratio < 0.16	48	Cylinder: −0.17	Different ablation ratios did not have a significant impact on astigmatism correction. However, a larger ablation ratio led to a greater increase in postoperative total higher-order aberrations (t HOAs) and spherical aberrations
				Ablation depth ratio 0.16–0.21	52	Cylinder: −0.18 ± 0.1	
				Ablation depth ratio > 0.21	48	Cylinder: −0.19 ± 0.1	

Abbreviations: AE, angle of error; CI, correction index; DV, difference vector; IOS, index of success; ME, magnitude of error.

## Data Availability

The data supporting the findings of this study are available from the corresponding author upon reasonable request.

## Supporting Information

The detailed search strategy for the PubMed database, conducted on January 1, 2024, is available in the supporting information.
