# Supplementary material for: Factors Influencing Astigmatic Correction Using Small-Incision Lenticule Extraction: A Systematic Review and Meta-Analysis
Source: J Ophthalmol. 2025 Nov 30;2025:5518587. doi: 10.1155/joph/5518587 (PMC12682452; doi:10.1155/joph/5518587)
Supplement: Supporting Information — Additional supporting information can be found online in the Supporting Information section. [file 5518587.f1.docx]

**Supplementary Materials 1：**

Search strategy for PubMed (January 1, 2024)

| **#** | **Searches** |
| --- | --- |
| #1 | Lenticule extraction OR Small-incision lenticule extraction OR Small incision lenticule extraction OR Refractive lenticule extraction OR Femtosecond lenticule extraction OR Femtosecond laser small incision lenticule extraction OR Small incision corneal lens extraction OR corneal stromal lenticule OR Refractive lenticule extraction OR SMILE |
| #2 | “Astigmatism” [MeSH] |
| #3 | Astigmatism* OR Cylind* OR Vector analysis OR Astigmia |
| #4 | #2 OR #3 |
| #5 | #1 AND #4 |
